# Gut microbiota in type 2 diabetes mellitus: mechanistic links between dysbiosis, insulin resistance, and chronic low-grade inflammation

**DOI:** 10.3389/fendo.2026.1856667

**Published:** 2026-06-02

**Authors:** Yi Chen, Danru Jin, Xue Han, Xiaoting Liu, Yisi Liu, Li Wang

**Affiliations:** 1 Department of Preventive Treatment of Disease, Affiliated Hospital of Liaoning University of Traditional Chinese Medicine, Liaoning, China; 2 Department of Cardiology I, The Affiliated Hospital of Liaoning University of Traditional Chinese Medicine, Liaoning, China

**Keywords:** bile acid signalling, branched-chain amino acid, gut dysbiosis, insulin resistance, microbial metabolites, short-chain fatty acids, type 2 diabetes mellitus

## Abstract

It is becoming more well acknowledged that type 2 diabetes mellitus (T2DM) is a metabolic and inflammatory condition linked to microbiota that involves interrelated disruptions in intestinal integrity, immune control, and insulin signalling. Butyrate-producing bacteria, such as Faecalibacterium prausnitzii and Roseburia spp., are reduced in gut dysbiosis in type 2 diabetes, whereas opportunistic Gram-negative pathobionts that cause endotoxemia and mucosal inflammation proliferate. Increased intestinal permeability makes it easier for lipopolysaccharide (LPS) to translocate and activate the TLR4/MyD88/IKKβ/NF-κB pathway. This increases the production of TNF-α, IL-6, MCP-1, and IL-1β, which disrupt insulin signalling by serine phosphorylation of IRS-1 and subsequent inhibition of PI3K/Akt/GLUT4 function. Concurrently, JNK and NLRP3 inflammasome pathway activation increases oxidative stress, caspase-1 activation, and inflammatory β-cell damage. Simultaneously, decreased microbial-derived short-chain fatty acid synthesis impairs GPR41/GPR43- and HDAC-mediated signalling, which in turn affects AMPK activation, mitochondrial function, and enteroendocrine release of GLP-1 and PYY. FXR-FGF19 and TGR5-cAMP signalling are further disrupted by altered bile acid biotransformation, which encourages hepatic gluconeogenesis, fat buildup, and insulin resistance. Moreover, dysregulated branched-chain amino acid metabolism and overactivation of the mTOR/S6K1 pathway lead to chronic low-grade inflammation and metabolic rigidity. When taken as a whole, these interrelated microbiota-host signalling pathways are significant mechanistic contributors to the pathophysiology of type 2 diabetes and new treatment targets.

## 1 Introduction

A major global public health concern of the twenty-first century, T2DM affects about 537 million adults globally and is expected to become much more common in the ensuing decades (1). Decreased insulin responsiveness in skeletal muscle, liver, and adipose tissue, followed by progressive pancreatic β-cell failure, are the hallmarks of this essentially compromised insulin signalling condition. At the molecular level, insulin resistance results from abnormalities in phosphatidylinositol 3-kinase (PI3K) signalling, insulin receptor substrate (IRS) activation, downstream Akt-mediated glucose uptake, and hepatic gluconeogenesis inhibition. Despite being significant upstream stressors, obesity and a surplus of nutrients do not adequately account for the chronic inflammatory tone and signalling abnormalities seen in T2DM, indicating the existence of other metabolic modulators that persistently disrupt insulin function (2). Recent developments in metagenomics and targeted metabolomics have revealed that metabolites generated from the gut microbiota are important modulators of host insulin signalling pathways (3). T2DM is reliably linked to functional changes in microbial metabolic output, namely pathways involved in the fermentation of carbohydrates, the transformation of Bile acids (BAs), the metabolism of amino acids, and the creation of endotoxins, rather than isolated shifts in microbial taxa. These metabolites penetrate systemic circulation and immediately interact with host metabolic and immunological signalling networks, functioning as mechanistic bridges between gut dysbiosis and insulin resistance (1).

SCFAs are a key regulatory node that connects insulin sensitivity and microbial metabolism. Through a number of convergent mechanisms, butyrate and propionate improve the action of insulin. These mechanisms include activating G-protein-coupled receptors (GPR41 and GPR43), stimulating the secretion of GLP-1, increasing mitochondrial oxidative capacity, and suppressing pro-inflammatory kinase signalling. Decreased SCFA availability in T2DM is caused by a decrease in the number of bacteria that produce SCFA. This leads to weaker enteroendocrine signalling, poor peripheral tissue glucose uptake, and heightened vulnerability to inflammatory interference with insulin signalling. Bile acid biotransformation, which is regulated by the gut microbiota, adds another level of metabolic control (4). The composition and signalling strength of circulating BAs are changed by microbial deconjugation and 7α-dehydroxylation, which in turn affects the activities of TGR5 and FXR. In T2DM, dysregulated bile acid signalling has been associated with elevated inflammatory signalling, decreased insulin sensitivity, and enhanced hepatic glucose production (HGP). This underscores the significance of microbial regulation of bile acid pools in maintaining metabolic homeostasis. Unlike these defence mechanisms, dysbiosis encourages the buildup of compounds that directly disrupt insulin signalling. Increased BCAAs disrupt IRS phosphorylation by activating mTOR and stress kinase pathways, and TMAO has been linked to metabolic inflammation and decreased glucose tolerance (5). Most importantly, gut barrier integrity disturbance brought on by dysbiosis raises systemic exposure to LPS, which causes metabolic endotoxemia. LPS triggers signalling cascades that are dependent on toll-like receptors 4 and NF-κB and JNK, which in turn prevent insulin signalling by serine phosphorylating IRS proteins. This persistent low-grade inflammatory state, which is marked by increased levels of interleukin-6, interleukin-1β, and tumour necrosis factor-α, increases β-cell failure and maintains insulin resistance. Crucially, inflammatory signalling further modifies the synthesis of metabolites and the composition of microorganisms, creating a feedback loop that connects insulin resistance, metabolite imbalance, ongoing inflammation, and dysbiosis (6). The present evidence is summarized in this narrative review to identify metabolite-driven pathways by which gut microbiota disruption leads to insulin resistance and low-grade inflammation in T2DM. This review seeks to elucidate mechanistic targets for microbiome-informed treatment interventions by concentrating on molecular signalling pathways instead of descriptive microbial alterations.

### 1.1 Literature search and analysis strategy

This review was conducted via a thorough literature search of PubMed, Scopus, Web of Science, and Google Scholar. Search phrases utilised included short-chain fatty acids, bile acids, lipopolysaccharide, type 2 diabetes mellitus, gut microbiota, dysbiosis, insulin resistance, inflammation, and microbial metabolites. Priority was given to articles published mostly between 2015 and 2026, with an emphasis on mechanistic research, clinical research, and meta-analyses pertinent to gut microbiota and type 2 diabetes.

## 2 Gut microbiota in health and T2DM

### 2.1 Microbiota composition & functional potential

In healthy individuals, gut microbiota functions as a dynamic metabolic and immunological system, supporting the processing of nutrients, preserving the integrity of the epithelial barrier, aiding in the metabolism of bile acids, and regulating immune function. Firmicutes and Bacteroidetes make up the majority of this ecosystem, with Actinobacteria, Verrucomicrobia, and Proteobacteria also having some participation. Eubiosis is characterized by high microbial diversity, ecological stability, and functional redundancy, all of which promote the continuous synthesis of advantageous metabolites in spite of environmental changes (7). Butyrate-producing bacteria, including Faecalibacterium prausnitzii, Roseburia spp., Eubacterium rectale, and members of the Lachnospiraceae and Ruminococcaceae families, are among the most important beneficial taxa. These bacteria are crucial for preserving colonocyte energy metabolism and controlling mucosal inflammation (8).

On the other hand, gut microbial dysbiosis, which includes disturbed community structure, changed metabolite synthesis, and compromised host-microbe signalling, is consistently linked to type 2 diabetes mellitus (T2DM). Certain taxonomic trends are consistent across clinical cohorts despite inter-study variation. According to several studies, there is an increase in opportunistic or pro-inflammatory taxa like Escherichia-Shigella, Ruminococcus gnavus, Collinsella, Blautia, and certain Proteobacteria along with a decrease in SCFA-producing genera like Faecalibacterium, Roseburia, Subdoligranulum, Anaerostipes, and Eubacterium (9, 10). According to a recent systematic review, people with type 2 diabetes and healthy controls consistently differed in their beta diversity. Akkermansia, Bifidobacterium, Bacteroides, Roseburia, Faecalibacterium, and Prevotella were found to have a negative relationship with T2DM, while certain genera, such as Lactobacillus, Escherichia–Shigella, Enterococcus, Subdoligranulum, and Fusobacterium, were found to have positive relationships (9). Blautia showed a positive correlation with T2DM, but Prevotella_9 and Odoribacter were found to be inversely linked in another investigation (11).

These findings have been reinforced by recent metagenomic studies that have shown functional dysbiosis rather than just compositional dysbiosis. Three main enterotypes—Bacteroides, Firmicutes, and Prevotella—were found using metagenomic nanopore sequencing, with the Firmicutes enterotype showing noticeably greater species richness and evenness. Functional changes in KEGG pathways, especially those related to homoacetogenesis, urea degradation, and amino acid breakdown (such as arginine metabolism), were linked to type 2 diabetes. Predictive modelling also revealed an increase in Absiella species and a decrease in Blautia and Enterococcus faecium. & Eubacterium limosum as possible T2DM microbiological markers (12). In a different research, Bacteroidaceae, Lachnospiraceae, Blautia, and Lachnospiraceae_FCS020_group were enriched in the gut microbial profiles of T2DM patients, but Streptococcaceae were more prevalent in the controls. Significant changes in bile acid metabolism, glucose homeostasis, fatty acid metabolism, and branched-chain amino acid biosynthesis pathways were identified by metabolomic analyses (3). There is growing evidence that dysbiosis and complications from diabetes are related. A study found that patients with microvascular problems and type 2 diabetes had significantly different gut microbial diversity and composition, with 3727 OTUs and unique group-specific OTU distributions. Compared to controls, the complication group had lower levels of Gammaproteobacteria, Bacilli, and Verrucomicrobia, and higher relative abundances of Clostridia and Negativicutes. Several significantly changed KEGG pathways between groups were also found using functional prediction (Tax4Fun) (13). In another investigation, Bacteroidales, Prevotellaceae, and Lachnospiraceae were more prevalent in the gut microbiota of DM patients with myocardial infarction than in DM-only individuals. Significant variations in KEGG orthologs units and pathways, such as ABC transporters and quorum sensing, were found by functional analysis (14). Additional biological plausibility has been added by mechanistic research. Bacterial DPP4-like enzymes have been found to be enriched in T2DM microbiomes, especially in Parabacteroides and Porphyromonas. These enzymes can break down incretin hormones like GLP-1 and GIP, which could affect glucose regulation (15). Subtype-specific correlations between gut microbiota and adult-onset T2DM symptoms (SIDD, SIRD, MOD, and MARD) were found using Mendelian randomization analysis. According to validation analysis, Holdemania and Catus were linked to higher risks of SIRD and MOD, respectively, but Clostridia/Clostridiales were linked to lower MOD risk (16).

### 2.2 Individual variability and confounding factors

Even though T2DM is frequently associated with gut dysbiosis, the determination of universal microbial signatures is limited by significant inter-individual variations in microbiota composition (17). This variability is a reflection of the complex interplay between the gut microbiota and T2DM, both of which are influenced by lifestyle choices, environmental exposures, and host characteristics. It can be challenging to distinguish between microbial patterns specific to diabetes and obesity because obesity, a common comorbidity of T2DM, independently modifies metabolic function and microbial composition. Even with controlling for body mass index, residual confounding frequently remains due to shared metabolic processes. One of the most potent regulators of gut microbiota and a separate factor in determining metabolic health is dietary behaviour. Diminished microbial diversity and enrichment of pro-inflammatory taxa are linked to diets heavy in refined carbohydrates and saturated fats, while fibre-rich, plant-based, and fermented foods maintain metabolically advantageous profiles and bacteria that produce SCFAs (18). Cross-sectional investigations make it difficult to draw conclusions about causality because diet affects both microbiota composition and the risk of T2DM. Thus, longitudinal methods are crucial for establishing temporal correlations and detecting early microbial changes that occur before the onset of disease. According to available data, gut dysbiosis and type 2 diabetes have a reciprocal rather than a one-way link. A study discovered that Clostridium citroniae, C. bolteae, Tyzzerella nexilis, and Ruminococcus gnavus predicted incident T2DM over 15.8 years among 5,572 Finnish people without diabetes, suggesting a potential pre-disease contributing role of dysbiosis (19). On the other hand, another study showed that hyperglycaemia itself intestinal barrier failure through GLUT2-dependent epithelial transcriptional alterations and loss of tight and adherens junction integrity, promoting systemic exposure to microbial products (20). Additionally, long-term high-glucose exposure directly modifies intestinal barrier morphology and function in intestinal epithelial models (21). Together, these results imply that anomalies in the gut microbiota may play a role in the early onset of metabolic dysfunction, while chronic hyperglycaemia and the diabetes metabolic environment may later promote and maintain dysbiosis over time.

A further layer of complexity is introduced by the use of medication. According to recent research, antidiabetic medications themselves can alter the composition of the gut microbiota as well as metabolites derived from the microbiome (22). For example, a study demonstrated that short-term metformin in newly diagnosed T2DM reduced Bacteroides fragilis, elevated the bile acid glycoursodeoxycholic acid, and suppressed intestinal FXR signalling, indicating a beneficial microbiota–bile acid pathway (23). Additionally, it has been demonstrated that acarbose dramatically alters the microbial composition of the gut in people with type 2 diabetes, leading to a decrease in Bacteroides and an increase in Bifidobacterium, Lactobacillus, and Eubacterium. By raising the ratio of primary to secondary bile acids and circulating unconjugated bile acids, acarbose also changed microbial bile acid metabolism in treatment-naïve patients (24, 25). In high-fat-diet mice, DPP-4 inhibitors changed the structure of gut microbes, prevented 68.6% of genus-level changes caused by HFD, elevated Bacteroidetes, and improved succinate synthesis; FMT verified that the DPP-4i-modified microbiota improved glucose tolerance (26). Further research indicates that SGLT2 inhibitors and GLP-1 receptor agonists may also affect the makeup of gut microbes. Liraglutide, semaglutide, and empagliflozin reduced inflammatory or LPS-associated bacteria while increasing SCFA-associated and metabolically beneficial taxa, according to experimental and clinical studies. However, there is still less proof of consistent microbiota-derived metabolite changes than for metformin and acarbose. Therefore, when assessing microbiota composition and metabolite profiles in type 2 diabetes, exposure to antidiabetic medicines should be regarded as a significant complicating factor (27–29).

Biological gender may also lead to inter-individual variability, as investigations have showed sex-specific variations in gut microbiota makeup and their interaction with sex hormones, which are connected to differences in metabolic illness vulnerability (30, 31). Additional variation results from cultural practices, age, ethnicity, geographic location and lifestyle factors such as physical activity level (sedentary *vs*. active) all of which influence baseline gut microbiota composition (i.e., the composition of the microbial community before the commencement of a disease or treatment) (32). Collectively, these factors highlight the necessity of using integrative analytical techniques and meticulously regulated, well-phenotyped groups when assessing the role of the microbiome in the pathophysiology of T2DM.

## 3 Microbial metabolites

### 3.1 Short-chain fatty acids

**Figure 1:**
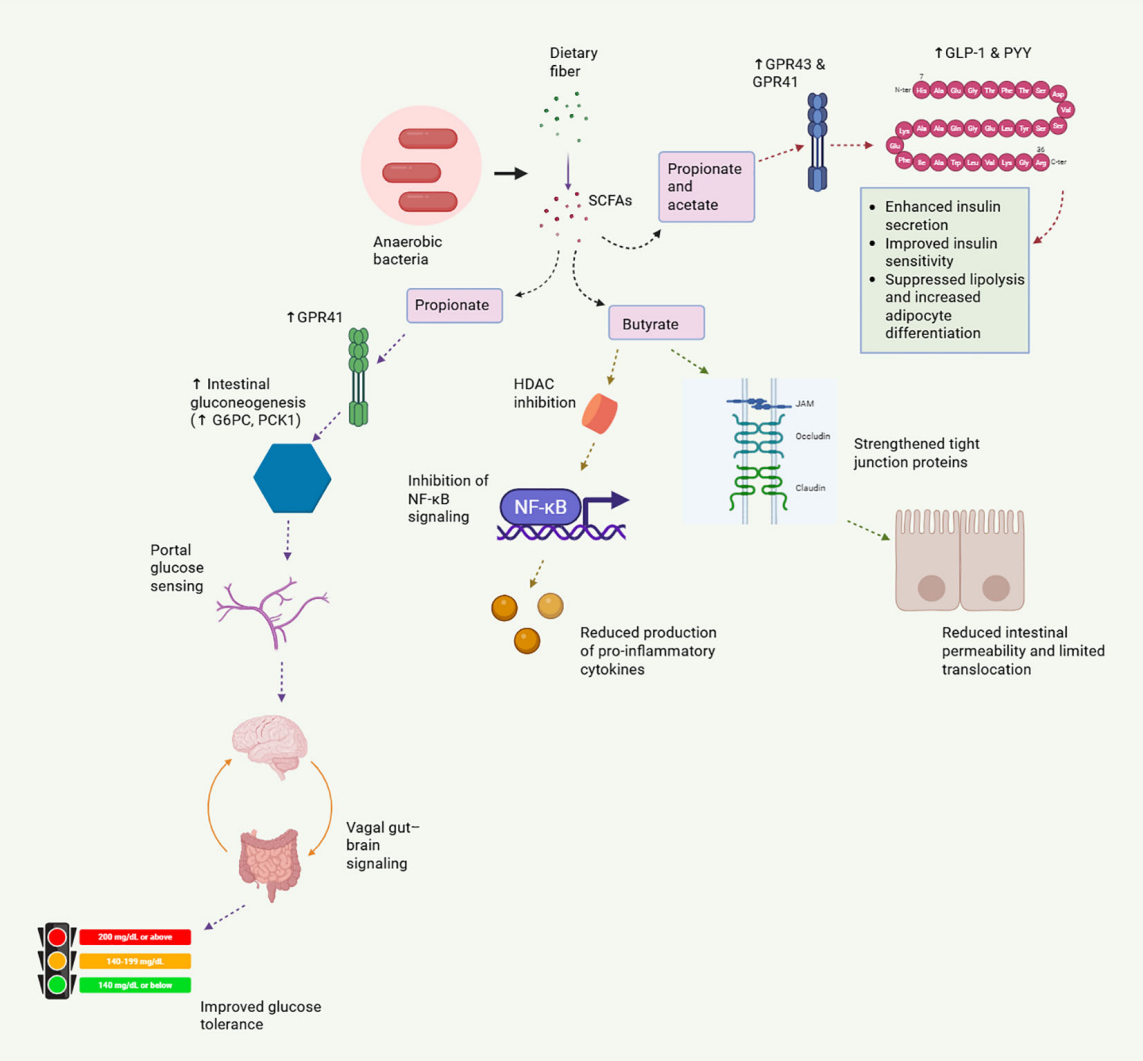
Metabolic and immune regulatory effects of gut microbiota–generated SCFAs.

SCFAs primarily acetate, propionate, and butyrate are key metabolic products of gut bacterial fermentation of dietary fibres and resistant starches. These metabolites are produced by specific anaerobic bacteria, including *Bacteroides* spp. that generate propionate and *F. prausnitzii*, *Roseburia* spp., *Eubacterium rectale*, and *Anaerostipes* spp., which produce butyrate. The abundance of SCFA is further influenced by cooperative “cross-feeding” interactions between microorganisms (33). Alterations in these SCFA-generating taxa are frequently seen in T2DM, and they are linked to decreased levels of SCFA in the blood and faeces, which indicates compromised microbial fermentative function. According to a study, a decreased prevalence of type 2 diabetes was linked to higher faecal contents of total SCFAs, acetate, and butyrate (11). According to a another investigation, T2DM was substantially correlated with reduced faecal acetate levels (34). Mechanistically, SCFAs increase insulin sensitivity, promote GLP-1 and PYY production, and inhibit inflammatory signalling via activating FFAR2/FFAR3 and GPR109A. Additionally, butyrate reduces endotoxin translocation and systemic inflammation by fortifying epithelial tight junctions and preserving the integrity of the intestinal barrier (35, 36) (**Figure 1**).

But since faecal SCFA concentrations do not accurately reflect systemic availability or microbial production alone, results pertaining to SCFA should be evaluated cautiously. Numerous factors, such as dietary fibre consumption, intestinal transit duration, epithelial absorption, microbial cross-feeding, and host metabolic condition, affect SCFA levels. Because of this, some research reports lower SCFA levels in T2DM, whereas other studies find inconsistent or metabolite-specific relationships across various groups. As a result, it is more accurate to think of SCFAs as dynamic, context-dependent signalling molecules whose metabolic effects rely on both host physiology and microbial composition.

### 3.2 Bile acid transformation

BAs are amphipathic molecules derived from cholesterol that serve as endocrine controllers of glucose, lipid, and energy metabolism in addition to its role in lipid emulsification. Hepatocytes produce primary BAs, such as chenodeoxycholic acid (CDCA) and cholic acid (CA), which are then converted to taurine or glycine and released into the intestine. Although most are reabsorbed in the terminal ileum through enterohepatic circulation, some make their way to the colon where they are converted into secondary BAs, such as deoxycholic acid (DCA) and lithocholic acid (LCA), by gut microbes that express the enzymes BSH and 7α-dehydroxylase (37). T2DM is linked to altered bile acid profiles and a decreased number of microbial genes contributing to bile acid metabolism, according to clinical and metagenomic findings. A human cohort-based multi-omics investigation revealed that higher circulating bile acids, a shift toward secondary bile acids, and changed glycine-to-taurine conjugation ratios are associated with worse glycaemic control in people with prediabetes and type 2 diabetes, although obesity alone had no discernible effect. Additionally, the study showed that these bile acid variations were strongly linked to changes in the microbiome and enhanced microbial metabolic interactions, such as polyamine and carbohydrates cross-feeding, highlighting a robust microbiota–bile acid–host metabolic axis (38). Another multi-omics investigation found changes in the content of faecal bile acids, which were marked by an enrichment of pathways associated with primary bile acid production and cholesterol metabolism (39). These changes affect signalling via Takeda G-protein receptor 5 (TGR5) and farnesoid X receptor (FXR), which control the metabolism of lipids and glucose. Research indicates that dysregulated FXR signalling increases hepatic gluconeogenesis and insulin resistance, whereas defective TGR5 activation decreases GLP-1 production (40).

Various investigations on T2DM continue to yield conflicting results for primary, secondary, conjugated, and unconjugated bile acid pools. A mechanistic link between microbial bile acid metabolism and glucose regulation is supported by the FXR and TGR5 signalling pathways, but these connections are heavily impacted by variables like obesity, hepatic steatosis, diet, drugs, and variations in microbial bile salt hydrolase activity. Therefore, rather than being a constant cause of insulin resistance, microbiota-related bile acid remodelling is better understood as a context-dependent metabolic regulator.

### 3.3 Amino acid–derived metabolites

Branched-chain amino acids. In type 2 diabetes mellitus (T2DM), amino acid-derived metabolites produced by the gut microbiota are crucial for controlling inflammation, insulin sensitivity, and general metabolic homeostasis. Among these metabolites, branched-chain amino acids (BCAAs), especially leucine, isoleucine, and valine, have been most frequently associated with metabolic dysfunction. Regardless of traditional markers like obesity, elevated circulation levels of BCAAs particularly have been repeatedly linked to an increased risk of type 2 diabetes (T2DM) (41, 42). However, a recent Mendelian randomization investigation revealed no causal relationship between valine, leucine, or isoleucine and the risk of type 2 diabetes. Instead, genetically predicted T2DM was strongly correlated with elevated levels of all three BCAAs in the blood, indicating that BCAAs are primarily metabolic biomarkers rather than direct causative agents (43). In support of this, metagenomic investigations have revealed strain-specific changes in the gut microbiome in people with type 2 diabetes, namely elevated BCAA production pathways in species like Prevotella copri (44). T2DM-related gut dysbiosis is typified by increased microbial BCAA production and decreased breakdown, which results in their buildup. Mechanistically, BCAAs activate mTORC1, a crucial regulator of nutrient sensing; nevertheless, persistent activation inhibits insulin signalling by phosphorylating IRS-1. Furthermore, inadequate BCAA catabolism leads to the accumulation of hazardous intermediates, such as acylcarnitines and branched-chain keto acids, which impair mitochondrial function and worsen oxidative stress and insulin resistance. Emerging data indicated that metabolic dysfunction is more strongly correlated with decreased BCAA catabolic capacity than with absolute levels (45).

However, there is still debate regarding the function of BCAAs in type 2 diabetes. Insulin resistance and incidence type 2 diabetes are regularly linked to elevated BCAAs, however there is growing evidence that this relationship may be reciprocal rather than strictly causal. Early metabolic disorders such as obesity, altered hepatic metabolism, and gut dysbiosis may raise systemic BCAA concentrations whereas chronically high BCAAs may worsen insulin resistance through defective catabolism, mitochondrial stress, and persistent mTORC1/S6K1 activation.

### 3.4 Tryptophan metabolism

Tryptophan metabolism mediated by the gut bacteria is a significant immunometabolic process implicated in the pathophysiology of type 2 diabetes. Dietary tryptophan is converted by gut bacteria into indole derivatives, such as indole aldehyde, indole-3-propionic acid, and indole-3-acetic acid (46). These metabolites support tight-junction integrity, anti-inflammatory host-microbe communication, and mucosal tolerance primarily through aryl hydrocarbon receptor signalling. In a study, higher levels of blood indolepropionic acid (IPA) were linked to better insulin secretion, lower inflammation, increased dietary fibre intake, and a lower risk of type 2 diabetes. The potential impact of IPA on reducing the incidence of type 2 diabetes may be mediated via the interaction between dietary fibre consumption and inflammation or by the direct impact of IPA on β-cell activity (46). On the other hand, dysregulated tryptophan metabolism may promote toxic compounds like indoxyl sulphate, which is linked to metabolic inflammation, oxidative stress, and endothelial dysfunction (47).

### 3.5 Other metabolites

A number of additional bioactive substances produced by gut microbiota are linked to metabolic dysfunction in type 2 diabetes mellitus (T2DM). Succinate functions as a metabolic signalling molecule that is context-dependent. Increased circulating succinate has been linked to metabolic dysfunction and inflammatory activation in obesity and type 2 diabetes, while microbiota-derived succinate may promote glucose homeostasis in physiological conditions (48, 49). The microbial metabolism of dietary choline and L-carnitine produces trimethylamine N-oxide (TMAO), which is linked to an elevated risk of cardiovascular disease in people with type 2 diabetes. Although its direct effects on glucose metabolism are still unknown, it encourages atherosclerosis through inflammatory activation, foam cell production, and endothelial dysfunction (50, 51). Since blood levels are heavily impacted by food, renal function, hepatic metabolism, and cardiovascular comorbidities, the pathogenic role of TMAO in type 2 diabetes is still not fully known. Even while TMAO has been shown in experiments to affect β-cell activity and glucose tolerance, the majority of the present evidence is associative rather than causative.

On the other hand, advantageous phenolic metabolites like urolithins and equol, which have anti-inflammatory and antioxidant qualities and may improve insulin sensitivity and mitochondrial function, are produced by microbial metabolism of dietary polyphenols (52, 53). These effects differ based on the composition of each person’s gut flora. Furthermore, many vitamins that support metabolic processes—such as vitamin K and B vitamins—are produced by gut microorganisms and may be diminished in dysbiosis. Hydrogen sulfide created by sulfate-reducing bacteria has concentration-dependent effects; high concentrations damage the integrity of the intestinal barrier and cause inflammation (54). Another gut-microbiota metabolite that results from the breakdown of phenylalanine is phenylacetylglutamine (PAGln), which has drawn interest due to its connection to cardiometabolic risk. According to recent research, PAGln affects host cellular responses via interacting with adrenergic receptor signalling, especially β2-adrenergic pathways. Endothelial dysfunction and reduced vascular repair ability have been linked to increased circulating PAGln levels in type 2 diabetes (55) (**Table 1**).

**Table 1:** Gut microbiota–derived metabolites and their mechanistic contributions to T2DM.

Category	Metabolite	Key producing taxa/pathways	Primary host targets	Mechanistic impact on T2DM	References
SCFA	Butyrate	*F. prausnitzii*, *Roseburia* spp.; acetyl-CoA and butyryl-CoA:acetate CoA-transferase pathways	GPR43, GPR109A; colonocytes	Strengthens the gut barrier integrity, inhibits NF-κB–driven inflammation, enhances peripheral insulin sensitivity; significantly lowered in T2DM	(36)
SCFA	Propionate	*Bacteroides* spp., *Veillonella*; succinate, acrylate, and propanediol pathways	GPR41, enteroendocrine L-cells	Increases the release of GLP-1 and regulates hepatic gluconeogenesis; lowers faecal levels linked to insulin resistance	(56)
Bile acids	DCA, LCA	*Lactobacillus* spp., *Clostridium* spp.; bile salt hydrolase activity and 7α-dehydroxylation	FXR, TGR5 receptors	Impaired bile acid signalling disrupts glucose homeostasis, decreases incretin secretion, and exacerbates hepatic insulin resistance	(57)
Endotoxin	Lipopolysaccharide (LPS)	Structural outer-membrane component of Gram-negative bacteria (*Enterobacteriaceae*); elevated systemic exposure following barrier failure	CD14/TLR4/MyD88/NF-κB axis	Causes metabolic endotoxemia, persistent low-grade inflammation, macrophage invasion of adipose tissue, and insulin resistance	(58)
Amino acids	Leucine, Isoleucine, Valine	*Prevotella copri, Bacteroides vulgatus;* BCAA production, and alleviated host BCAA catabolism	mTORC1/S6K1, IRS-1, mitochondria	Associated with metabolic dysregulation, insulin resistance, and mitochondrial stress	(59)
Choline/carnitine metabolites	TMAO	*Clostridium* spp., *Desulfovibrio* spp., and Enterobacteriaceae convert dietary choline and L-carnitine into trimethylamine (TMA) through processes controlled by CutC/D and CntA/B, then hepatic FMO3 oxidation to TMAO	β-cells, vascular endothelium, inflammatory pathways	Encourages insulin resistance, oxidative damage, and systemic inflammation; increased plasma levels in T2DM	(50)
Tryptophan metabolites	Indole-3-propionic acid	*Clostridium sporogenes* and other indole-producing bacteria; microbial tryptophan metabolism	AhR, intestinal epithelial cells	Keeps the gut barrier intact and lowers inflammation; decreased circulating levels linked to T2DM chance	(60)
Other	Succinate	Dysbiotic microbiota Bacteroides spp. and Prevotella spp.; altered carbohydrate fermentation pathways	SUCNR1, liver, immune cells	Serves as a context-dependent metabolic signal; long-term increased succinate in obesity and type 2 diabetes may lead to inflammation and metabolic dysregulation, whereas microbiota-derived succinate may enhance glucose homeostasis via intestinal gluconeogenesis.	(48, 49)

## 4 Intestinal barrier dysfunction: enabling systemic exposure

The intestinal barrier is a multilayered, intricate structure that controls luminal contents’ selective transit while preventing dangerous microbial products from spreading throughout the body. The epithelial monolayer, which is sealed by tight junction proteins including occludin, claudins, and zonula occludens (ZO-1) that regulate paracellular permeability, is largely responsible for its integrity. The mucus barrier, which is mainly made up of mucin glycoproteins like MUC2, covers this layer and functions as a biochemical and physical barrier to keep commensal microorganisms and the epithelial surface apart (61). Goblet cells, Paneth cells, and antimicrobial peptides, such as defensins, all contribute to this defence. The barrier maintains immunological tolerance and allows nutrition absorption under physiological settings, but it is nevertheless very sensitive to microbial and metabolic disruptions. The generation of short-chain fatty acids (SCFAs), especially butyrate, is a major way that gut bacteria contribute to the preservation of epithelial integrity. Type 2 diabetes mellitus (T2DM) dysbiosis causes SCFA deficit, which impairs epithelial energy metabolism and tight junction stability (9). Simultaneously, the mucus layer may be eroded by an increase of opportunistic pathobionts or mucin-degrading bacteria (such as Akkermansia muciniphila under dysregulated environments), further exposing the epithelium. Enhanced intestinal permeability, sometimes known as “leaky gut,” results from barrier breakdown and permits luminal components to move into the systemic circulation. Among these, the lipopolysaccharide (LPS) found in the cell walls of Gram-negative bacteria is especially important. Increased circulating LPS known as metabolic endotoxemia, has been repeatedly associated with low-grade inflammation and insulin resistance in T2DM (62).

## 5 Immune activation and chronic low-grade inflammation

**Figure 2:**
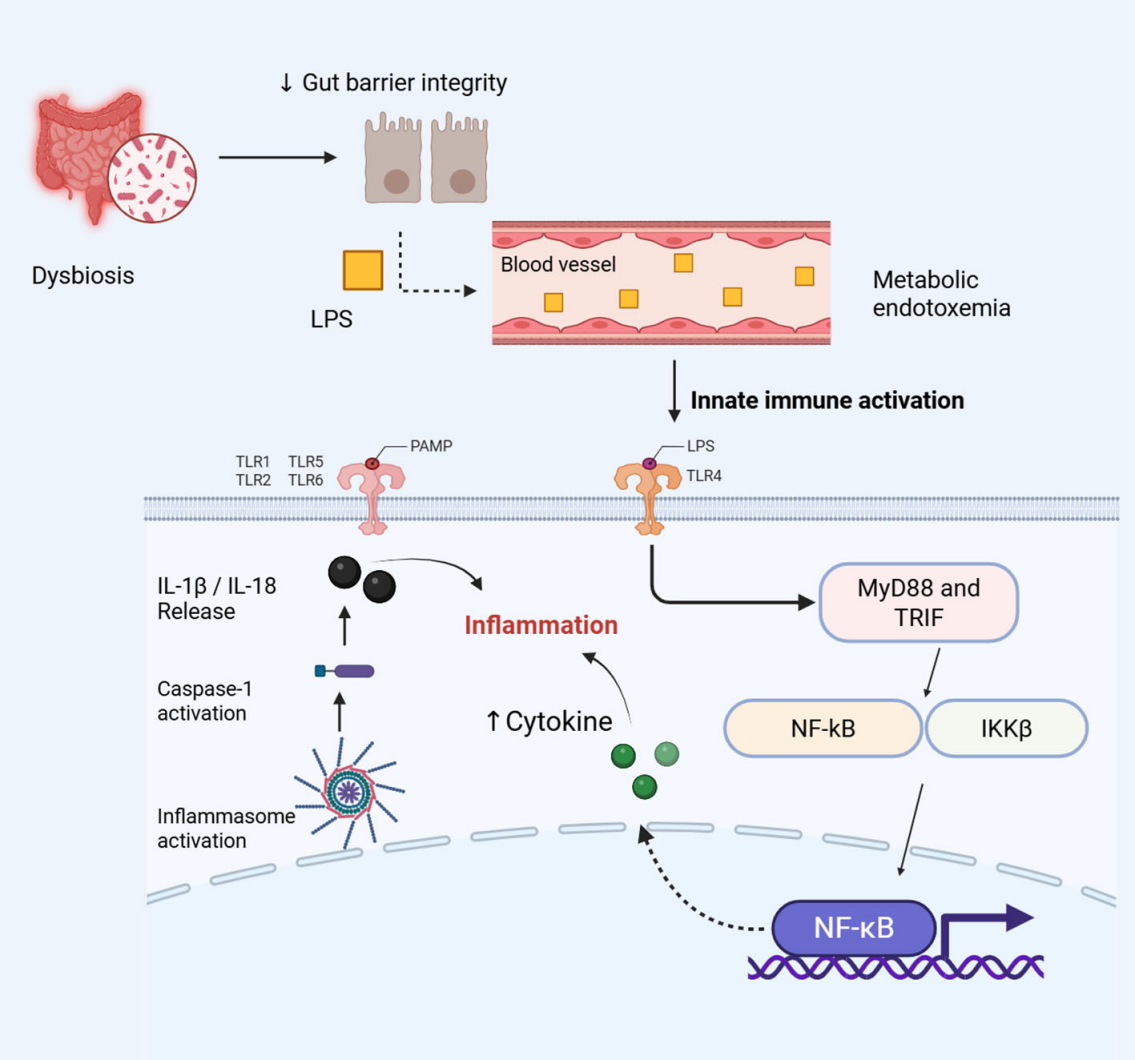
Dysbiosis-induced metabolic endotoxemia stimulates innate immune system and inflammation.

LPS, peptidoglycan fragments, flagellin, bacterial DNA, and microbial extracellular vesicles can translocate to intestinal immune cells and peripheral metabolic organs such as skeletal muscle, adipose tissue, liver, and pancreatic islets due to a higher intestinal permeability. The engagement of TLR2, TLR4, TLR5, NOD1, and NOD2 signalling networks by these microbial elements leads to stimulation of NF-κB, AP-1, JNK, and IKKβ via MyD88 and RIP2 (63). By means of serine phosphorylation of IRS-1 and decreased PI3K/Akt function, this inflammatory reaction promotes the production of TNF-α, IL-6, MCP-1, IL-1β, and iNOS, which impede insulin signalling (58, 64). The activation of the NLRP3 inflammasome is an additional important route. NLRP3 in macrophages, hepatocytes, adipose tissue, and pancreatic islets is activated by microbial endotoxins in T2DM in conjunction with hyperglycaemia, saturated fatty acids, ceramides, mitochondrial ROS, and extracellular ATP (65). This results in triggering of caspase-1 and the generation of IL-1β and IL-18, which worsen β-cell stress, hepatic insulin resistance, and adipose inflammation. L-1β is especially important as it reduces β-cell proliferation, insulin secretion, and β-cell functional identity during metabolic stress situation (66) (**Figure 2**).

Inflammatory signals from the gut also modify the immunological activity of adipose tissue. Dysbiosis and Metabolic endotoxemia increase the migration of inflammatory monocytes into adipose tissue through MCP-1/CCR2, where they develop into pro-inflammatory M1-like macrophages. These macrophages form crown-like structures surrounding hypertrophic adipocytes and emit reactive oxygen species (ROS), iNOS, TNF-α, IL-6, and IL-1β. This inflammatory milieu increases circulating free fatty acids, encourages lipolysis, and causes insulin resistance in skeletal muscle, adipose tissue, and the liver (67).

Persistent metaflammation in type 2 diabetes is also influenced by adaptive immunological dysregulation. Dendritic cells and other antigen-presenting immune cells are triggered by dysbiotic microbial substances to release IL-12, IL-23, TNF-α, and IL-6. This promotes the development of pro-inflammatory Th1 and Th17 cells. Increased Th17 activity and IL-17 signalling enhance neutrophil recruitment, epithelial inflammation, adipose tissue dysfunction, and insulin resistance, thereby contributing to persistent metabolic inflammation (68, 69). Concurrently, butyrate-producing bacterial reduction decreases SCFA-mediated activation of Foxp3+ regulatory T cells via GPR43/GPR109A signalling and HDAC suppression. Decreased Treg activity promotes chronic low-grade inflammation and an ongoing Th17/Treg imbalance in T2DM by weakening immunological tolerance mediated by TGF-β and IL-10 (70). All together, gut dysbiosis stimulates metaflammation via a variety of interrelated innate and adaptive immunological pathways. These inflammatory networks come together to maintain long-term exposure to cytokines in adipose tissue, liver, skeletal muscle, pancreatic islets, and vascular endothelium. This disrupts insulin signalling, exacerbates metabolic dysfunction, and speeds up the progression of β-cell failure in T2DM (**Table 2**).

**Table 2:** Host immune-inflammatory connecting dysbiosis to inflammation and insulin resistance in T2DM.

Host pathway	Dysbiosis-related upstream signal	Key cellular/molecular events	Effect on inflammation and insulin resistance	References
TLR2/TLR4–MyD88–IRAK/TRAF6–NF-κB/MAPK axis	Extracellular vesicles, bacterial DNA, lipoproteins, and LPS translocation following enhanced epithelial permeability	MyD88-dependent NF-κB, AP-1, JNK, p38 MAPK, and IKKβ signalling activation in endothelial cells, hepatocytes, adipocytes, and macrophages	TNF-α, IL-6, MCP-1, IL-1β, and iNOS are elevated; PI3K/Akt insulin signalling is weakened and IRS-1 serine phosphorylation is encouraged.	(58, 63)
NOD1/NOD2–RIP2–NF-κB axis	Translocation of bacterial peptidoglycan motifs (iE-DAP, MDP) resulting from enhanced gut permeability and dysbiotic microbiota	NOD1/NOD2-mediated RIP2 polyubiquitination increases the generation of IL-6, TNF-α, IL-1β, and chemokines by activating NF-κB and MAPK signalling	Increases metabolic inflammation and impairs insulin signalling in the liver, skeletal muscle, and adipose tissue.	(71)
NLRP3 inflammasome–caspase-1–IL-1β/IL-18 axis	Endotoxin priming in conjunction with saturated fatty acids, ceramides, mitochondrial ROS, extracellular ATP, and hyperglycaemia	NLRP3 formation, ASC recruitment, caspase-1 stimulation, and maturation of IL-1β/IL-18	Maintains hepatic and adipose inflammation, exacerbates insulin resistance, and leads to inflammatory stress in β-cells.	(72)
JNK/IKKβ stress-kinase pathway	Long-term exposure to lipotoxic signals, endotoxins, oxidative stress, and cytokines	JNK and IKKβ stimulation in skeletal muscle, adipose tissue, liver, and β-cells	Effectively blocks insulin receptor signalling by IRS-1 serine phosphorylation and decreasing Akt stimulation	(73)
MCP-1/CCR2 monocyte recruitment pathway	Endotoxemia brought on by dysbiosis and the discharge of chemokines from immune cells and adipocytes	Inflammatory monocyte recruitment reliant on CCR2; increase of M1-like macrophages in adipose tissue	Increases the release of free fatty acids, ROS, lipolysis, TNF-α, IL-6, and IL-1β, all of which worsen systemic insulin resistance.	(67, 74)
Th1/Th17 polarization axis	Dysbiosis-induced stimulation of dendritic cells and antigen-presenting cells	Elevated levels of IL-12, IL-23, IL-6, and TNF-α promote Th1/Th17 differentiation and IL-17 signalling	Encourages neutrophil recruitment, adipose and epithelial inflammation, and chronic metaflammation associated with insulin resistance.	(69)
Treg insufficiency/Th17–Treg imbalance	Absence of tolerogenic microbiota-derived immune control	Decreased Foxp3+ Treg activity accompanied by reduced TGF-β and IL-10 signalling	Reduces immunological tolerance, permits persistent low-grade inflammation, and hence reduces insulin sensitivity.	(70)

## 6 Downstream development of insulin resistance

**Figure 3:**
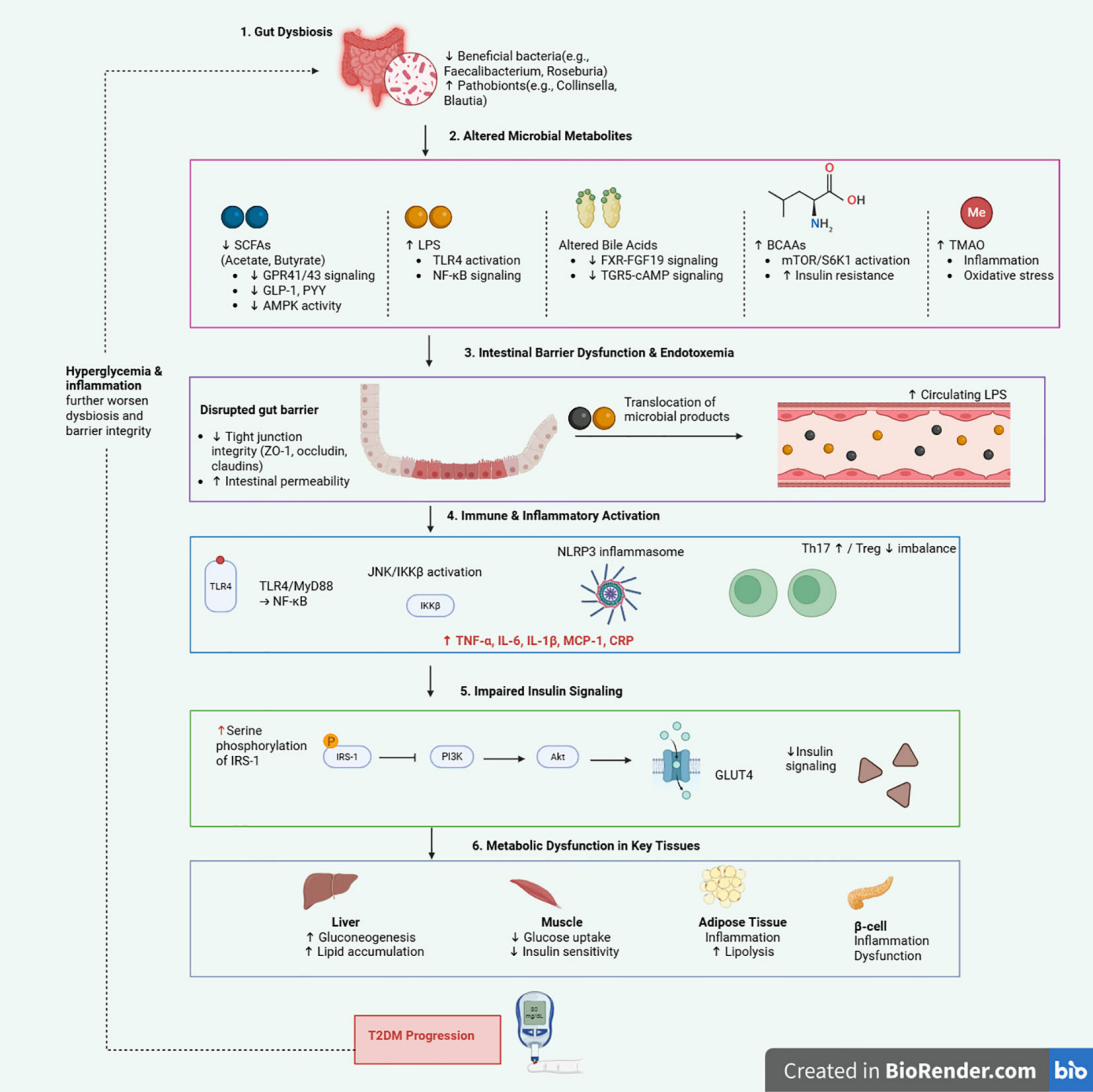
Integrated microbiota–immune pathways involved in T2DM.

Insulin resistance develops as a result of chronic low-grade inflammation, which gradually disturbs metabolic homeostasis in type 2 diabetes. In metabolically active tissues, persistent inflammatory signalling modifies intracellular insulin signalling pathways by activating stress kinases such as IκB kinase (IKKβ) and c-Jun N-terminal kinase (JNK). Insulin receptor substrate (IRS) proteins are serine phosphorylated by these stress kinases, which reduces their capacity to transmit insulin signals (73). The downstream phosphoinositide 3-kinase (PI3K)–Akt signalling, which is necessary for glucose absorption and metabolic control, is weakened by this disturbance. Insulin response is consequently significantly decreased in peripheral tissues. The effects of insulin resistance on metabolism are tissue-specific. In the liver, disrupted insulin signalling fails to inhibit gluconeogenesis, resulting in higher hepatic glucose production. In skeletal muscle, decreased Akt stimulation restricts translocation of glucose transporter type 4 (GLUT4), lowering glucose absorption (75). Insulin resistance interferes with adipokine secretion in adipose tissue, resulting in increased leptin and decreased adiponectin, as well as increased lipolysis and the release of free fatty acids. Ectopic fat accumulation and inflammation are made worse by these lipids. Initially, pancreatic β-cells adapt by producing hyperinsulinemia; however, long-term exposure to glucolipotoxicity and inflammatory stress causes increasing β-cell malfunction and ultimately a decrease in insulin production, which signals the onset of overt diabetes (4) (**Figure 3**).

## 7 Translation and therapeutic interventions

### 7.1 Dietary strategies

A key component of microbiota-directed strategies for enhancing metabolic control in people with T2DM is dietary intervention. In addition to the macronutrient composition alone, diet influences the gut ecosystem through changes in the supply of fermentable substrates, microbial metabolic outputs, and host–microbe signalling processes that control inflammation, the integrity of the gut barrier, and glucose homeostasis. A growing number of clinical and mechanistic studies are demonstrating the close relationship between diet-induced changes in microbial function and alterations in insulin sensitivity and systemic metabolic health (76). In T2DM, fermentable dietary fibre is the most evaluated nutritional modulator of the gut microbiota. Non-digestible carbohydrates like inulin, β-glucans, arabinoxylans, and resistant starch evade breakdown in the small intestine and are fermented by colonic bacteria into SCFAs, primarily acetate, propionate, and butyrate. Fiber-dense diets can improve insulin sensitivity and lower HbA1c and fasting glucose in T2DM populations, according to clinical feeding trials. These consequences coincide with rises in faecal SCFAs and a shift toward saccharolytic fermentation (77).

For example, a study found that an isoenergetic, fibre-rich diet changed the gut microbiota of people with T2DM by lowering metabolically detrimental taxa and selectively increasing important bacteria that produce SCFA. Increased abundance and diversity of these beneficial microbes were associated with better glycaemic control, partly via enhanced GLP-1 secretion (78). The high-fibre diet helped participants with T2DM maintain better glucose homeostasis in a different investigation. High-fibre diet raised the proportions of advantageous gut microbes, while lowering the amount of opportunistic pathogens such as Desulfovibrio and Klebsiella. This was demonstrated by the rise in levels of Lactobacillus, Bifidobacterium, and Akkermansias (79). A meta-analysis of T2DM patients, dietary fibre supplementation, specifically soluble fibre, significantly improved fasting insulin, fasting glucose, HbA1c, and insulin resistance when compared control interventions, however, the included studies exhibited significant heterogeneity and were primarily short-term trials (80).

A whole-diet strategy that promotes a microbiome that is metabolically efficient is the Mediterranean diet pattern. This pattern, which is low in ultra-processed foods and high in fruits, vegetables, legumes, whole grains, nuts, and extra virgin olive oil, is linked to increased microbial diversity and enrichment of taxa like butyrate producers and *A. muciniphila*. In patients with metabolic disorders, randomized interventions show that Mediterranean-style diets enhance insulin sensitivity and lower inflammatory biomarkers (81). According to a separate study, following a Mediterranean diet enhanced the Prevotella/Bacteroides ratio and gut microbial diversity in people with T2DM. Early microbiota changes were linked to better insulin resistance and fasting glucose levels. HbA1c and HOMA-IR decreased with long-term adherence (82). According to a meta-analysis, Mediterranean diet interventions constantly increased microbial diversity with enrichment of beneficial taxa like *Roseburia* species and *A. muciniphila*, and they also substantially decreased HbA1c, LDL cholesterol, and triglycerides (83). On the other hand, Western-style diets high in saturated fats and refined carbohydrates decrease microbial diversity, encourage pro-inflammatory taxa, and compromise gut barrier function, all of which lead to metabolic endotoxemia and insulin resistance (84). Even though dietary treatments have shown quick microbiota remodelling and metabolic benefits, the variation in study designs highlights the necessity for standardized protocols to promote clinical translation.

### 7.2 Probiotics and next-generation biotherapeutics

Probiotics provide a focused approach to alter the composition and function of the gut microbiota in T2DM. Lactobacillus and Bifidobacterium strains have been studied the most, and several of these species have been connected to better metabolic and inflammatory profiles in people with T2DM. Mechanistically, certain strains improve the integrity of the intestinal barrier by increasing the production of tight junction proteins and mucins, which lowers the transfer of endotoxins. The generation of short-chain fatty acids, bile acid metabolism, and enteroendocrine signalling—specifically, the secretion of glucagon-like peptide-1 (GLP-1) are additional impacts that contribute to better metabolic regulation (85). A systematic review and meta-analysis of randomized controlled trials showed that probiotic intake can result in modest improvements in fasting glucose, HbA1c, and insulin resistance indices as well as decreases in circulating inflammatory markers (86, 87). According to a recent systematic review and meta-analysis, microbiome-targeted treatments, especially substrate-matched synbiotics and multi-strain probiotics, are useful supplements for enhancing glycaemic control. These benefits are probably mediated through increased microbial metabolite production (88). A randomized controlled experiment repeatedly revealed that BL21 supplementation, in conjunction with metformin, significantly lowered HbA1c and exhibited positive trends in insulin resistance and fasting glucose when compared to placebo. Along with good safety and tolerability, these results were accompanied by favourable regulation of the gut microbiota, which was marked by an increase in Bifidobacterium and Faecalibacterium and a decrease in pathogenic taxa (89).

Emerging next-generation biotherapeutics made from commensals linked to metabolic health have drawn more and more attention. In preclinical models, *A. muciniphila* is reliably reduced in obesity and T2DM, has shown improvements in gut barrier integrity, insulin sensitivity, and glucose tolerance (90). Additionally, a proof-of-concept human study found that in adults who were overweight or insulin-resistant, pasteurized *A. muciniphila* intake decreased insulinemia and increased insulin sensitivity (91). While *F. prausnitzii*, a prominent butyrate producer with anti-inflammatory qualities, is also being studied, its stringent anaerobic growth requirements make it difficult to develop as a live probiotic, which has sparked interest in using bacterial components or metabolites as substitutes (92). Despite encouraging results, there are still significant restrictions. The sustainability of metabolic effects is limited because probiotic strains frequently show only temporary colonization and little long-term integration into the resident microbiota. Additionally, the wide heterogeneity in strain selection, dosing tactics, and formulation types, restricts comparability among trials and hampers the establishment of standardized therapy protocols. Host-specific variables such as baseline microbial composition and metabolic phenotype further influence treatment response, highlighting the necessity of carefully monitored, strain-specific studies and precision-based methods to maximize clinical efficacy (**Table 3**).

**Table 3:** Clinical effects of therapeutic interventions in T2DM.

Intervention	Study design	Reported clinical effect size	Evidence strength	Key limitations	Key references
Soluble dietary fiber	Systematic review and meta-analysis of RCTs	HbA1c −0.66%; FPG (10 g: − 0.80 mmol/, > 10g: −1.02 mmol/L); HOMA-IR WMD − 1.27	Moderate–high	Variability in the type, frequency, or quantity of fibre consumption; primarily short-term trials	(80)
Mediterranean-style diet	Systematic review/meta-analysis of intervention trials	HbA1c −0.18%, LDL-cholesterol −0.10 mmol/L, and triglycerides −0.20 mmol/L	Moderate	Short interventions, small sample sizes, and significant variation in baseline metabolic traits and dietary regimens	(83)
Probiotics	Grade-assessed systematic review and dose-response meta-analysis	FBS −13.27 mg/dL; HbA1c −0.44%; fasting insulin −1.33 μIU/mL; HOMA-IR −0.95	Low-moderate	Significant variation in probiotic dosage, duration, species/strain selection, and overall quality of the evidence	(86, 87)
Probiotics, prebiotics and synbiotics	GRADE-assessed systematic review and meta-analysis	FPG −16.57 mg/dL; HbA1c −0.44%; insulin SMD −0.37; HOMA-IR	Moderate	Significant variation among treatments, inconsistent strains and dosages, and little ethnic diversity	(93)
Fecal microbiota transplantation (FMT)	Systematic review and meta-analysis	PBG −0.51 mmol/L, HOMA-IR −2.73, FPG −0.94 mmol/L	Low	Donor-dependent variability, small sample sizes, brief follow-up periods, a dearth of RCTs, and inconsistent transplantation procedures	(94)

### 7.3 Postbiotics and metabolite-based interventions

A durable and secure microbiota-based treatment approach for T2DM is postbiotics, which are characterized as inactivated bacteria and their bioactive components. Specifically, short-chain fatty acids like butyrate have been linked to enhanced gut barrier function, decreased systemic inflammation, and better insulin sensitivity (37). According to an experimental investigation, butyrate supplementation reduced diabetic endotoxemia in db/db mice by reestablishing the makeup of the gut microbiota and preserving the integrity of the intestinal epithelial barrier (95).

Propionate-targeted treatments, including inulin-propionate ester, have been shown to increase insulin sensitivity, decrease visceral adiposity, and positively regulate hormones linked to appetite. For example, inulin–propionate ester (IPE) dramatically enhanced insulin sensitivity comparable to inulin, according to a randomized crossover trial. Additionally, IPE decreased systemic inflammation (IL-8) and caused specific alterations in gut microbiota and metabolism, indicating a function for colonic propionate in glucose homeostasis (96). Other microbial metabolites, such as urolithins and indole derivatives, exhibit possible metabolic advantages associated with cellular function and the decrease of oxidative stress (97). Despite encouraging results, clinical translation is still hampered by low bioavailability and inconsistent dosage.

### 7.4 Fecal microbiota transplantation

The most extensive method of modifying gut microbial ecosystems is faecal microbiota transplantation (FMT), which involves receiving faecal material from metabolically healthy donors. Unlike single-strain probiotics, FMT allows for the complete restoration of microbial diversity and functional capacity, providing a mechanistic approach to combat low-grade inflammation and insulin resistance linked to dysbiosis in T2DM. Although FMT is well-established for recurring infections caused by *Clostridium difficile*, its use in metabolic disorders is still in the experimental stage (98). Proof-of-concept investigations in metabolic syndrome and T2DM have shown that lean donor FMT can temporarily enhance insulin sensitivity, enhance faecal butyrate levels, and elevate the relative abundance of SCFA-producing taxa (13, 99).

The Effectiveness of this approach is greatly influenced by factors like the donor microbiota makeup, recipient baseline microbial ecology, metabolic phenotype, concurrent medications (such as metformin or antibiotics), the delivery method, and the frequency of dosing. This variation is a reflection of the significant variation in treatment response between studies. “Super-donors,” whose microbial populations provide superior metabolic functionality, have been discovered; however, there is still ongoing research to define predictive microbial features (100). FMT might work mechanistically by boosting signalling mediated by BAs and SCFA, restoring microbial diversity, strengthening the integrity of the intestinal barrier, and reducing systemic inflammation. There are still issues with clinical viability, such as inconsistent donor screening procedures and differences in transplantation techniques (such as colonoscopic, nasoenteric, or oral capsule distribution), which could affect results. Unintentional metabolic or immunological impacts, possible pathogen transmission, and gastrointestinal adverse events are all safety concerns, furthermore, there is still a dearth of long-term safety data in populations with metabolic diseases. Variability in therapeutic response, the absence of standardized techniques, and safety issues, such as possible pathogen transfer, limit clinical applicability. Reproducibility and safety may be enhanced by standardizing donor selection and creating specific microbial consortia.

### 7.5 Precision microbiome medicine

Precision microbiome medicine reflects an emerging approach for optimizing the management of T2DM by customizing therapies to each patient’s traits. This method incorporates host genetics, metabolic status, and functional profiles acquired from the microbiome, to increase therapeutic efficacy and reduce side effects. Targeted interventions are made possible by the identification of dysbiosis patterns through microbiome profiling using 16S rRNA gene sequencing or shotgun metagenomics (101). People who are enriched in LPS-generating bacteria might need methods to improve intestinal barrier function, while those with low levels of SCFA-producing bacteria might benefit more from high-fibre diets, prebiotics, or SCFA-producing probiotics. Combining metabolomic data allows for functional readouts of microbial activity, which enhance compositional analyses and make customized treatment planning easier.

Personalized dietary and microbiological interventions are made possible by predictive modelling and machine learning techniques. The PREDICT study showed that postprandial glucose responses can be accurately predicted by baseline microbiome composition, clinical, and lifestyle factors (102). This allows for the creation of personalized dietary recommendations. The gut microbiota affects the pharmacokinetics and pharmacodynamics of medications like metformin, which affects the effectiveness of treatment, according to pharmacomicrobiomics. By combining metagenomics, metabolomics, metatranscriptomics, and host omics, multi-omics integration provides systems-level understanding of microbiota-host relationships and helps identify possible biomarkers and treatment targets (102). These methods encourage a change from broad-based treatment plans to tailored, microbiome-informed therapies, which may enhance metabolic outcomes and glycaemic control in T2DM. Precision microbiome therapy has great potential, but there are many translational obstacles to overcome, such as high cost, restricted access to multi-omics technologies, and a lack of standardised analytical frameworks for interpreting microbiome data. The creation of affordable sequencing platforms and therapeutically useful biomarkers is crucial to overcoming these obstacles.

## 8 Future directions

Future research should focus more on proving causation by integrating methods like controlled experimental systems (such gnotobiotic models), longitudinal cohort designs, and Mendelian randomisation. These tactics have recently reinforced the evidence that certain microbial taxa and their metabolites are associated with the onset of T2DM (103). Simultaneously, more human-relevant platforms, such as intestine organoids and microphysiological systems, should be used to more effectively simulate host-microbiome interactions under controlled conditions, enabling accurate study of metabolite-driven signalling processes. Furthermore, the combination of single-cell RNA sequencing with spatial transcriptomics is essential for capturing cellular heterogeneity and revealing cell-type-specific responses to microbial metabolites in metabolic tissues, eventually overcoming the inherent constraints of bulk-level analysis. Additionally, multi-omics integration (metagenomics, metabolomics, and host transcriptomics) in longitudinal studies should be preferred to determine temporal and causal links between dysbiosis, metabolite shifts, and progression from insulin resistance to overt T2DM (3, 17).

To reduce inter-study variability and improve reproducibility in both clinical and experimental contexts, microbiome research methodologies including sample techniques, sequencing technology, and bioinformatic workflows must be standardised. Crucially, future research on the microbiome should focus on functional characterisation at the strain and gene levels rather than just general taxonomic descriptions. According to a new large cross-cohort metagenomic analysis, microbial signatures linked to type 2 diabetes are more meaningful at the strain and functional pathway level than at the genus level. Inter-individual metabolic variability and disease aetiology may be influenced by functional changes related to quorum sensing, horizontal gene transfer, and glucose metabolism (44). The structure and function of microbial communities can now be thoroughly characterised thanks to developments in high-resolution sequencing and multi-omics technologies. This makes it easier to identify microbial pathways that are related to illness and host-microbe interactions. Investigating microbial enzyme activity as a therapeutic and mechanistic target is another new avenue. Increased gut microbiota-derived DPP4-like enzyme activity, especially Parabacteroides merdae-associated DPP4-like activity that can inactivate GLP-1 and GIP *in vivo*, has been shown in T2DM. Future microbiome research should quantify microbial protease activity, incretin breakdown capacity, and interaction with DPP4 inhibitor or GLP-1 receptor agonist therapy in addition to identifying changed taxa (15).

The precise modification of microbial genes and metabolic processes is made possible by emerging microbiome engineering techniques, especially CRISPR-based and metagenomic editing platforms. These techniques should be further developed for functional validation and specific therapeutic applications (104). Concurrently, to increase the accuracy and scalability of microbiome-directed approaches, real-time microbiome monitoring tools and targeted delivery systems (such as encapsulated metabolites, engineered consortia, and controlled-release formulations) must be developed (105).

Precision medicine techniques that combine microbiome profiles with clinical phenotypes—such as obesity, MASLD, and cardiovascular complications—should be used in future clinical research because these variables significantly influence T2DM heterogeneity and therapy response variability. Crucially, rather than addressing type 2 diabetes as a single, homogeneous metabolic disease, future research should focus on complication-specific microbiome profiling. Strong correlations between albuminuria and certain microbial taxa, like Sellimonas intestinalis, Eggerthellales sp., and Ellagibacter isourolithinifaciens, as well as metabolites like imidazole propionate and trigonelline, were found in the latest deep shotgun metagenomic and plasma metabolomic analyses. These results endorse the developing idea of microbiome-based risk stratification for diabetic kidney disease and underscore the increasing significance of gut–organ interaction networks in diabetes complications (106).

Furthermore, to thoroughly assess the effectiveness and long-term stability of microbiome-targeted therapies like probiotics, postbiotics, and faecal microbiota transplantation, well-powered, long-duration randomised controlled trials using standardised endpoints such as HbA1c, insulin sensitivity, and inflammatory biomarkers—are evidently needed (103). Combined therapy approaches that align microbiome modification with well-established medicines, such as metformin or incretin-based therapies, should be methodically investigated to identify possible synergistic metabolic effects. Instead of relying solely on generic microbiota manipulation, future medicinal development should advance toward planned microbiome engineering methodologies. Compared to traditional probiotic formulations, engineered bacterial strains and defined microbial consortia that can provide metabolically advantageous compounds, decrease detrimental microbial enzymatic activity, or restore incretin signalling may offer more therapeutic specificity. In experimental T2DM models, engineered Clostridium butyricum expressing GLP-1 has recently reduced blood glucose levels, controlled dyslipidaemia, and improved hepatic impairment, offering proof-of-concept for intended microbial drug-delivery systems in metabolic illnesses (107). Additionally, stratified techniques that utilize baseline microbiome composition and functional capability should be prioritized to improve tailored treatment outcomes and lower inter-individual variability (108).

## 9 Conclusion

There is growing evidence that the dysbiosis of the gut microbiota contributes to T2DM in a mechanistically relevant way by influencing insulin signalling, microbial metabolite production, and persistent, low-grade inflammation. Changed microbial metabolic output including decreased availability of SCFAs, irregular bile acid signalling, greater flux of BCAAs, and greater exposure to endotoxins, directly impairs insulin responsiveness and encourages inflammatory activation across important metabolic tissues. The two main mechanisms that connect dysbiosis to long-term impairment of insulin receptor signalling are intestinal barrier dysfunction and metabolic endotoxemia. All of these results point to a model where metabolites produced by the microbiota serve as vital bridges linking environmental exposures to host metabolic dysfunction. Although microbiome-focused therapies have therapeutic promise, clinical result heterogeneity highlights the need for better patient stratification, standardized procedures, and mechanistic resolution. Integrating clinical, metabolic, and microbiological data will be essential for future advancements in defining causal pathways and directing precision techniques. This framework must be developed further in order to convert microbiome research into practical approaches for managing and preventing T2DM.

## References

[1] Hossain MJ Al-Mamun M Islam MR . Diabetes mellitus, the fastest growing global public health concern: Early detection should be focused. Health Sci Rep. (2024) 7:e2004. doi: 10.1002/hsr2.2004 38524769 PMC10958528

[2] Galicia-Garcia U Benito-Vicente A Jebari S Larrea-Sebal A Siddiqi H Uribe KB . Pathophysiology of type 2 diabetes mellitus. Int J Mol Sci. (2020) 21:6275. doi: 10.3390/ijms21176275 32872570 PMC7503727

[3] Morsy Y Shafie NS GP2324 consortium . Integrative analysis of gut microbiota and metabolic pathways reveals key microbial and metabolomic alterations in diabetes. Sci Rep. (2025) 15:30686. doi: 10.1038/s41598-025-09328-w 40841806 PMC12371060

[4] Pham NHT Joglekar MV Wong WKM Nassif NT Simpson AM Hardikar AA . Short-chain fatty acids and insulin sensitivity: A systematic review and meta-analysis. Nutr Rev. (2024) 82:193–209. doi: 10.1093/nutrit/nuad042 37290429 PMC10777678

[5] Huang H Chen H Yao Y Lou X . Branched-chain amino acids supplementation induces insulin resistance and pro-inflammatory macrophage polarization via INFGR1/JAK1/STAT1 signal pathway. Mol Med (Cambridge Mass). (2024) 30:149. doi: 10.1186/s10020-024-00894-9 39267003 PMC11391606

[6] Obeagu EI . Unraveling the connection: Inflammatory markers and diabetes mellitus pathogenesis. Medicine. (2026) 105:e47338. doi: 10.1097/MD.0000000000047338 41578487 PMC12851792

[7] Bidell MR Hobbs ALV Lodise TP . Gut microbiome health and dysbiosis: A clinical primer. Pharmacotherapy. (2022) 42:849–57. doi: 10.1002/phar.2731 36168753 PMC9827978

[8] Ojala T Kankuri E Kankainen M . Understanding human health through metatranscriptomics. Trends Mol Med. (2023) 29:376–89. doi: 10.1016/j.molmed.2023.02.002 36842848

[9] Chong S Lin M Chong D Jensen S Lau NS . A systematic review on gut microbiota in type 2 diabetes mellitus. Front Endocrinol. (2025) 15:1486793. doi: 10.3389/fendo.2024.1486793 39897957 PMC11782031

[10] Saiedi E Shapouri R Haghi F Zeighami H . Comparative analysis of gut microbiota composition in the fecal samples from type 2 diabetes mellitus patients and healthy individuals: A case control study. Iranian J Microbiol. (2025) 17:875–84. doi: 10.18502/ijm.v17i6.20354 41510046 PMC12777383

[11] Yang Y Yan J Li S Liu M Han R Wang Y . Efficacy of fecal microbiota transplantation in type 2 diabetes mellitus: A systematic review and meta-analysis. Endocrine. (2024) 84:48–62. doi: 10.1007/s12020-023-03606-1 38001323

[12] Dash NR Al Bataineh MT Alili R Al Safar H Alkhayyal N Prifti E . Functional alterations and predictive capacity of gut microbiome in type 2 diabetes. Sci Rep. (2023) 13:22386. doi: 10.1038/s41598-023-49679-w 38104165 PMC10725451

[13] Wang Y Jiang D Pan X Sun K Li T Cao X . Gut microbiota in T2DM patients with microvascular complications: A 16S rRNA sequencing study. Diabetes Metab Syndrome Obesity: Targets Ther. (2025) 18:373–81. doi: 10.2147/DMSO.S493720 39963193 PMC11831918

[14] Zhang H Zhai C Hu H Qian G Mao M . A metagenomic study of the gut microbiome in patients with type 2 diabetes mellitus and myocardial infarction. Acta Diabetologica. Advance online publication. doi: 10.1007/s00592-026-02648-x 41661278 PMC13219173

[15] Olivares M Hernández-Calderón P Cárdenas-Brito S Liébana-García R Sanz Y Benítez-Páez A . Gut microbiota DPP4-like enzymes are increased in type-2 diabetes and contribute to incretin inactivation. Genome Biol. (2024) 25:174. doi: 10.1186/s13059-024-03325-4 38961511 PMC11221189

[16] Ruan Z Liu J Zhao J . Causal associations between gut microbiota and type 2 diabetes mellitus subtypes: A mendelian randomization analysis. BMC Endocr Disord. (2025) 25:79. doi: 10.1186/s12902-025-01863-x 40122799 PMC11931760

[17] Mashal R Al-Muhanna A Khader S Khudair A Khudair A Butler AE . The role of the gut microbiome in type 2 diabetes mellitus. Int J Mol Sci. (2025) 26:11412. doi: 10.3390/ijms262311412 41373570 PMC12691723

[18] Nova E Gómez-Martinez S González-Soltero R . The influence of dietary factors on the gut microbiota. Microorganisms. (2022) 10:1368. doi: 10.3390/microorganisms10071368 35889087 PMC9318379

[19] Ruuskanen MO Erawijantari PP Havulinna AS Liu Y Méric G Tuomilehto J . Gut microbiome composition is predictive of incident type 2 diabetes in a population cohort of 5,572 Finnish adults. Diabetes Care. (2022) 45:811–8. doi: 10.2337/dc21-2358 35100347 PMC9016732

[20] Thaiss CA Levy M Grosheva I Zheng D Soffer E Blacher E . Hyperglycemia drives intestinal barrier dysfunction and risk for enteric infection. Science. (2018) 359:1376–83. doi: 10.1530/ey.15.12.8 29519916

[21] Dubois N Muñoz-Garcia J Heymann D Renodon-Cornière A . High glucose exposure drives intestinal barrier dysfunction by altering its morphological, structural and functional properties. Biochem Pharmacol. (2023) 216:115765. doi: 10.1016/j.bcp.2023.115765 37619641

[22] Zhang Q Hu N . Effects of metformin on the gut microbiota in obesity and type 2 diabetes mellitus. Diabetes Metab Syndrome Obesity: Targets Ther. (2020) 13:5003–14. doi: 10.2147/DMSO.S286430 33364804 PMC7751595

[23] Sun L Xie C Wang G Wu Y Wu Q Wang X . Gut microbiota and intestinal FXR mediate the clinical benefits of metformin. Nat Med. (2018) 24:1919–29. doi: 10.1038/s41591-018-0222-4 30397356 PMC6479226

[24] Gu Y Wang X Li J Zhang Y Zhong H Liu R . Analyses of gut microbiota and plasma bile acids enable stratification of patients for antidiabetic treatment. Nat Commun. (2017) 8:1785. doi: 10.1038/s41467-017-01682-2 29176714 PMC5702614

[25] Takewaki F Nakajima H Takewaki D Hashimoto Y Majima S Okada H . Habitual dietary intake affects the altered pattern of gut microbiome by acarbose in patients with type 2 diabetes. Nutrients. (2021) 13:2107. doi: 10.3390/nu13062107 34205413 PMC8235473

[26] Liao X Song L Zeng B Liu B Qiu Y Qu H . Alteration of gut microbiota induced by DPP-4i treatment improves glucose homeostasis. EBioMedicine. (2019) 44:665–74. doi: 10.1016/j.ebiom.2019.03.057 30922964 PMC6603491

[27] Deng X Zhang C Wang P Wei W Shi X Wang P . Cardiovascular benefits of empagliflozin are associated with gut microbiota and plasma metabolites in type 2 diabetes. J Clin Endocrinol Metab. (2022) 107:1888–96. doi: 10.1210/clinem/dgac210 35397165 PMC9202724

[28] Duan X Zhang L Liao Y Lin Z Guo C Luo S . Semaglutide alleviates gut microbiota dysbiosis induced by a high-fat diet. Eur J Pharmacol. (2024) 969:176440. doi: 10.1016/j.ejphar.2024.176440 38402930

[29] Shang J Liu F Zhang B Dong K Lu M Jiang R . Liraglutide-induced structural modulation of the gut microbiota in patients with type 2 diabetes mellitus. PeerJ. (2021) 9:e11128. doi: 10.7717/peerj.11128 33850659 PMC8019531

[30] Koliada A Moseiko V Romanenko M Lushchak O Kryzhanovska N Guryanov V . Sex differences in the phylum-level human gut microbiota composition. BMC Microbiol. (2021) 21:131. doi: 10.1186/s12866-021-02198-y 33931023 PMC8088078

[31] Santos-Marcos JA Mora-Ortiz M Tena-Sempere M Lopez-Miranda J Camargo A . Interaction between gut microbiota and sex hormones and their relation to sexual dimorphism in metabolic diseases. Biol Sex Differ. (2023) 14:4. doi: 10.1186/s13293-023-00490-2 36750874 PMC9903633

[32] Schlicht K Pape L Rohmann N Knappe C Epe J Geisler C . Physical activity induced alterations of gut microbiota in humans: A systematic review. BMC Sports Sci Med Rehabil. (2022) 14:122. doi: 10.1186/s13102-022-00513-2 35799284 PMC9264679

[33] Overby HB Ferguson JF . Gut microbiota-derived short-chain fatty acids facilitate microbiota:Host cross talk and modulate obesity and hypertension. Curr Hypertension Rep. (2021) 23:8. doi: 10.1007/s11906-020-01125-2 33537923 PMC7992370

[34] Puig R Rojo-López MI Julve J Castelblanco E Ponomarenko J Amézqueta S . Fecal short-chain fatty acids to predict prediabetes and type 2 diabetes risk: An exploratory cross-sectional study. Nutrients. (2025) 17:3003. doi: 10.3390/nu17183003 41010528 PMC12472725

[35] Murugesan R Kumar J Leela KV Meenakshi S Srivijayan A Thiruselvam S . The role of gut microbiota and bacterial translocation in the pathogenesis and management of type 2 diabetes mellitus: Mechanisms, impacts, and dietary therapeutic strategies. Physiol Behav. (2025) 293:114838. doi: 10.1016/j.physbeh.2025.114838 39922411

[36] Saban Güler M Arslan S Ağagündüz D Cerqua I Pagano E Berni Canani R . Butyrate: A potential mediator of obesity and microbiome via different mechanisms of actions. Food Res Int (Ottawa Ont). (2025) 199:115420. doi: 10.1016/j.foodres.2024.115420 39658184

[37] Shapiro H Kolodziejczyk AA Halstuch D Elinav E . Bile acids in glucose metabolism in health and disease. J Exp Med. (2018) 215:383–96. doi: 10.1084/jem.20171965 29339445 PMC5789421

[38] Schlicht K Pape L Rohmann N Knappe C Epe J Geisler C . Prediabetes and type 2 diabetes but not obesity are associated with alterations in bile acid related gut microbe-microbe and gut microbe-host community metabolism. Gut Microbes. (2025) 17:2474143. doi: 10.1080/19490976.2025.2474143 40045464 PMC11901388

[39] Wang Y Xu H Zhou X Chen W Zhou H . Dysregulated bile acid homeostasis: Unveiling its role in metabolic diseases. Med Rev (2021). (2024) 4:262–83. doi: 10.1515/mr-2024-0020 39135605 PMC11317083

[40] Kim H Fang S . Crosstalk between FXR and TGR5 controls glucagon-like peptide 1 secretion to maintain glycemic homeostasis. Lab Anim Res. (2018) 34:140–6. doi: 10.5625/lar.2018.34.4.140 30671099 PMC6333617

[41] Flores-Guerrero JL Osté MCJ Kieneker LM Gruppen EG Wolak-Dinsmore J Otvos JD . Plasma branched-chain amino acids and risk of incident type 2 diabetes: Results from the PREVEND prospective cohort study. J Clin Med. (2018) 7:513. doi: 10.3390/jcm7120513 30518023 PMC6306832

[42] Ramzan I Ardavani A Vanweert F Mellett A Atherton PJ Idris I . The association between circulating branched chain amino acids and the temporal risk of developing type 2 diabetes mellitus: A systematic review & meta-analysis. Nutrients. (2022) 14:4411. doi: 10.3390/nu14204411 36297095 PMC9610746

[43] Mosley JD Shi M Agamasu D Vaitinadin NS Murthy VL Shah RV . Branched-chain amino acids and type 2 diabetes: A bidirectional Mendelian randomization analysis. Obes (Silver Spring Md). (2024) 32:423–35. doi: 10.1002/oby.23951 38269471 PMC10827349

[44] Mei Z Wang F Bhosle A Dong D Mehta R Ghazi A . Strain-specific gut microbial signatures in type 2 diabetes identified in a cross-cohort analysis of 8,117 metagenomes. Nat Med. (2024) 30:2265–76. doi: 10.1038/s41591-024-03067-7 38918632 PMC11620793

[45] Biswas D Dao KT Mercer A Cowie AM Duffley L El Hiani Y . Branched-chain ketoacid overload inhibits insulin action in the muscle. J Biol Chem. (2020) 295:15597–621. doi: 10.1074/jbc.RA120.013121 32878988 PMC7667962

[46] Tuomainen M Lindström J Lehtonen M Auriola S Pihlajamäki J Peltonen M . Associations of serum indolepropionic acid, a gut microbiota metabolite, with type 2 diabetes and low-grade inflammation in high-risk individuals. Nutr Diabetes. (2018) 8:35. doi: 10.1038/s41387-018-0046-9 29795366 PMC5968030

[47] Wu Y Li T Chen B Sun Y Song L Wang Y . Tryptophan indole derivatives: Key players in type 2 diabetes mellitus. Diabetes Metab Syndrome Obesity: Targets Ther. (2025) 18:1563–74. doi: 10.2147/DMSO.S511068 40386349 PMC12083488

[48] De Vadder F Kovatcheva-Datchary P Zitoun C Duchampt A Bäckhed F Mithieux G . Microbiota-produced succinate improves glucose homeostasis via intestinal gluconeogenesis. Cell Metab. (2016) 24:151–7. doi: 10.1016/j.cmet.2016.06.013 27411015

[49] Sabadell-Basallote J Astiarraga B Castaño C Ejarque M Repollés-de-Dalmau M Quesada I . SUCNR1 regulates insulin secretion and glucose elevates the succinate response in people with prediabetes. J Clin Invest. (2024) 134:e173214. doi: 10.1172/JCI173214 38713514 PMC11178533

[50] DiNicolantonio JJ McCarty M OKeefe J . Association of moderately elevated trimethylamine N-oxide with cardiovascular risk: Is TMAO serving as a marker for hepatic insulin resistance. Open Heart. (2019) 6:e000890. doi: 10.1136/openhrt-2018-000890 30997120 PMC6443140

[51] Kong L Zhao Q Jiang X Hu J Jiang Q Sheng L . Trimethylamine N-oxide impairs β-cell function and glucose tolerance. Nat Commun. (2024) 15:2526. doi: 10.1038/s41467-024-46829-0 38514666 PMC10957989

[52] Hamaura K Murakami H Tamura A Matsuki K Sato E Tanabe J . Association between equol producers and type 2 diabetes mellitus among Japanese older adults. J Diabetes Invest. (2023) 14:707–15. doi: 10.1111/jdi.13995 36852538 PMC10119921

[53] Toney AM Fan R Xian Y Chaidez V Ramer-Tait AE Chung S . Urolithin A, a gut metabolite, improves insulin sensitivity through augmentation of mitochondrial function and biogenesis. Obes (Silver Spring Md). (2019) 27:612–20. doi: 10.1002/oby.22404 30768775

[54] Pichette J Gagnon J . Implications of hydrogen sulfide in glucose regulation: How H2S can alter glucose homeostasis through metabolic hormones. Oxid Med Cell Longevity. (2016) 2016:3285074. doi: 10.1155/2016/3285074 27478532 PMC4958482

[55] Huang L Ye X Ho CK Gao Y Wen D Sun J . Type 2 diabetes-associated phenylacetylglutamine induces deleterious inflammation cycle in myeloid cells through β2 adrenergic receptors and impedes wound healing. Advanced Sci (Weinheim Baden-Wurttemberg Germany). (2025) 12:e08205. doi: 10.1002/advs.202508205 40810653 PMC12622421

[56] Facchin S Calgaro M Savarino EV . Rethinking short-chain fatty acids: A closer look at propionate in inflammation, metabolism, and mucosal homeostasis. Cells. (2025) 14:1130. doi: 10.3390/cells14151130 40801563 PMC12346497

[57] Wang Y Lv B Liu N Tao S Dou J Li J . The mechanism of bile acid metabolism regulating lipid metabolism and inflammatory response in T2DM through the gut-liver axis. Heliyon. (2024) 10:e35421. doi: 10.1016/j.heliyon.2024.e35421 39229512 PMC11369409

[58] Fuke N Nagata N Suganuma H Ota T . Regulation of gut microbiota and metabolic endotoxemia with dietary factors. Nutrients. (2019) 11:2277. doi: 10.3390/nu11102277 31547555 PMC6835897

[59] Lo EKK Felicianna Xu JH Zhan Q Zeng Z El-Nezami H . The emerging role of branched-chain amino acids in liver diseases. Biomedicines. (2022) 10:1444. doi: 10.3390/biomedicines10061444 35740464 PMC9220261

[60] Sehgal R de Mello VD Männistö V Lindström J Tuomilehto J Pihlajamäki J . Indolepropionic acid, a gut bacteria-produced tryptophan metabolite and the risk of type 2 diabetes and non-alcoholic fatty liver disease. Nutrients. (2022) 14:4695. doi: 10.3390/nu14214695 36364957 PMC9653718

[61] Ghosh SS Wang J Yannie PJ Ghosh S . Intestinal barrier dysfunction, LPS translocation, and disease development. J Endocr Soc. (2020) 4:bvz039. doi: 10.1210/jendso/bvz039 32099951 PMC7033038

[62] Mazaheri-Tehrani S Rezaei F Heidari-Hasanabadi S Malakoutikhah M Amani-Beni R Arefian M . Serum lipopolysaccharide binding protein (LBP) and metabolic syndrome: A systematic review and meta-analysis. Diabetol Metab Syndrome. (2025) 17:268. doi: 10.1186/s13098-025-01847-w 40671146 PMC12269216

[63] Chen L Zhang L Hua H Liu L Mao Y Wang R . Interactions between toll-like receptors signaling pathway and gut microbiota in host homeostasis. Immun Inflammation Dis. (2024) 12:e1356. doi: 10.1002/iid3.1356 39073297 PMC11284964

[64] Chen S Xie M Liu Y . TLR2 promotes the progression of diabetes mellitus with atherosclerosis via activating NLRP3 inflammasome and MyD88/NF-κB signaling pathway. Sci Rep. (2025) 15:16348. doi: 10.1038/s41598-025-00843-4 40348852 PMC12065783

[65] Lebreton F Berishvili E Parnaud G Rouget C Bosco D Berney T . NLRP3 inflammasome is expressed and regulated in human islets. Cell Death Dis. (2018) 9:726. doi: 10.1038/s41419-018-0764-x 29941940 PMC6018156

[66] Sétula C Pensado-Evans I Scelza-Figueredo A Orellano MS Rodríguez-Valero I Spinedi E . IL-1β priming triggers an adaptive stress response that enhances pancreatic β-cell resilience to subsequent cytotoxic inflammatory insult. Cell Death Dis. (2025) 16:744. doi: 10.1038/s41419-025-08059-0 41120257 PMC12540857

[67] Morita Y Senokuchi T Yamada S Wada T Furusho T Matsumura T . Impact of tissue macrophage proliferation on peripheral and systemic insulin resistance in obese mice with diabetes. BMJ Open Diabetes Res Care. (2020) 8 (1):e001578. doi: 10.1136/bmjdrc-2020-001578 33087339 PMC7580054

[68] Huang X Liu M Gonzalez MV Debnath R Afzali H Choi Y . Diabetes exacerbates destructive inflammation by activating the CD137L-CD137 axis in dendritic and IL-17+ T cells. J Clin Invest. (2026) 136:e193289. doi: 10.1172/jci193289 41379565 PMC12867133

[69] Tao L Liu H Gong Y . Role and mechanism of the Th17/Treg cell balance in the development and progression of insulin resistance. Mol Cell Biochem. (2019) 459:183–8. doi: 10.1007/s11010-019-03561-4 31218568 PMC6679830

[70] Kibbie JJ Dillon SM Thompson TA Purba CM McCarter MD Wilson CC . Butyrate directly decreases human gut lamina propria CD4 T cell function through histone deacetylase (HDAC) inhibition and GPR43 signaling. Immunobiology. (2021) 226:152126. doi: 10.1016/j.imbio.2021.152126 34365090 PMC8478853

[71] Gulzar F Chhikara N Kumar P Ahmad S Yadav S Gayen JR . ER stress aggravates NOD1-mediated inflammatory response leading to impaired nutrient metabolism in hepatoma cells. Biochem Biophys Res Commun. (2024) 735:150827. doi: 10.1016/j.bbrc.2024.150827 39423570

[72] Elliott EI Miller AN Banoth B Iyer SS Stotland A Weiss JP . Cutting edge: Mitochondrial assembly of the NLRP3 inflammasome complex is initiated at priming. J Immunol (Baltimore Md 1950). (2018) 200:3047–52. doi: 10.4049/jimmunol.1701723 29602772 PMC5916517

[73] Alipourfard I Datukishvili N Mikeladze D . TNF-α downregulation modifies insulin receptor substrate 1 (IRS-1) in metabolic signaling of diabetic insulin-resistant hepatocytes. Mediators Inflammation. (2019) 2019:3560819. doi: 10.1155/2019/3560819 30863203 PMC6378771

[74] Lu B Huang L Cao J Li L Wu W Chen X . Adipose tissue macrophages in aging-associated adipose tissue function. J Physiol Sci. (2021) 71:38. doi: 10.1186/s12576-021-00820-2 34863096 PMC10717320

[75] Liu J Li F Yang L Luo S Deng Y . Gut microbiota and its metabolites regulate insulin resistance: Traditional Chinese medicine insights for T2DM. Front Microbiol. (2025) 16:1554189. doi: 10.3389/fmicb.2025.1554189 40177494 PMC11963813

[76] Nitzke D Czermainski J Rosa C Coghetto C Fernandes SA Carteri RB . Increasing dietary fiber intake for type 2 diabetes mellitus management: A systematic review. World J Diabetes. (2024) 15:1001–10. doi: 10.4239/wjd.v15.i5.1001 38766430 PMC11099360

[77] Tsitsou S Athanasaki C Dimitriadis G Papakonstantinou E . Acute effects of dietary fiber in starchy foods on glycemic and insulinemic responses: A systematic review of randomized controlled crossover trials. Nutrients. (2023) 15:2383. doi: 10.3390/nu15102383 37242267 PMC10223420

[78] Zhao L Zhang F Ding X Wu G Lam YY Wang X . Gut bacteria selectively promoted by dietary fibers alleviate type 2 diabetes. Sci (New York NY). (2018) 359:1151–6. doi: 10.1126/science.aao5774 29590046

[79] Chen L Liu B Ren L Du H Fei C Qian C . High-fiber diet ameliorates gut microbiota, serum metabolism and emotional mood in type 2 diabetes patients. Front Cell Infect Microbiol. (2023) 13:1069954. doi: 10.3389/fcimb.2023.1069954 36794003 PMC9922700

[80] Mao T Huang F Zhu X Wei D Chen L . Effects of dietary fiber on glycemic control and insulin sensitivity in patients with type 2 diabetes: A systematic review and meta-analysis. J Funct Foods. (2021) 82:104500. doi: 10.1016/j.jff.2021.104500 38826717

[81] Dayi T Ozgoren M . Effects of the Mediterranean diet on the components of metabolic syndrome. J Prev Med Hygiene. (2022) 63:E56–64. doi: 10.15167/2421-4248/jpmh2022.63.2S3.2747 36479500 PMC9710414

[82] Ismael S Silvestre MP Vasques M Araújo JR Morais J Duarte MI . A pilot study on the metabolic impact of Mediterranean diet in type 2 diabetes: Is gut microbiota the key? Nutrients. (2021) 13:1228. doi: 10.3390/nu13041228 33917736 PMC8068165

[83] Lauria F Formisano A Dello Russo M Quaglia C Giacco R Russo GL . Mediterranean diet, gut microbiota, and type 2 diabetes: A systematic review and meta-analysis of intervention trials. Nutrition Metabolism Cardiovasc Dis: NMCD. (2026) 36 (5):104433. doi: 10.1016/j.numecd.2025.104433 41381307

[84] Clemente-Suárez VJ Beltrán-Velasco AI Redondo-Flórez L Martín-Rodríguez A Tornero-Aguilera JF . Global impacts of Western diet and its effects on metabolism and health: A narrative review. Nutrients. (2023) 15:2749. doi: 10.3390/nu15122749 37375654 PMC10302286

[85] Latif A Shehzad A Niazi S Zahid A Ashraf W Iqbal MW . Probiotics: Mechanism of action, health benefits and their application in food industries. Front Microbiol. (2023) 14:1216674. doi: 10.3389/fmicb.2023.1216674 37664108 PMC10470842

[86] Ayesha IE Monson NR Klair N Patel U Saxena A Patel D . Probiotics and their role in the management of type 2 diabetes mellitus (short-term versus long-term effect): A systematic review and meta-analysis. Cureus. (2023) 15:e46741. doi: 10.7759/cureus.46741 38022046 PMC10631563

[87] Yao K Zeng L He Q Wang W Lei J Zou X . Effect of probiotics on glucose and lipid metabolism in type 2 diabetes mellitus: A meta-analysis of 12 randomized controlled trials. Med Sci Monitor Int Med J Exp Clin Res. (2017) 23:3044–53. doi: 10.12659/msm.902600 28638006 PMC5491138

[88] Shalbaf N Sadeghi S Homaee S Saberian F . Probiotics, prebiotics, synbiotics, and FMT for glycemic control: A systematic review of clinical efficacy and mechanistic readouts in type 2 diabetes and related dysglycemia. Metab Open. (2025) 28:100419. doi: 10.1016/j.metop.2025.100419 41321404 PMC12664430

[89] Zhu C Liu Y Chen Y Wang Z Gao Y Xu F . Probiotic supplementation with Bifidobacterium longum subsp. longum BL21 improves glycemic control and modulates gut microbiota in type 2 diabetes: A randomized controlled trial. Food Sci Nutr. (2026) 14:e71437. doi: 10.1002/fsn3.71437 41523285 PMC12784097

[90] Liu E Ji X Zhou K . Akkermansia muciniphila for the prevention of type 2 diabetes and obesity: A meta-analysis of animal studies. Nutrients. (2024) 16:3440. doi: 10.3390/nu16203440 39458436 PMC11510203

[91] Depommier C Everard A Druart C Plovier H Van Hul M Vieira-Silva S . Supplementation with Akkermansia muciniphila in overweight and obese human volunteers: A proof-of-concept exploratory study. Nat Med. (2019) 25:1096–103. doi: 10.1038/s41591-019-0495-2 31263284 PMC6699990

[92] Maioli TU Borras-Nogues E Torres L Barbosa SC Martins VD Langella P . Possible benefits of Faecalibacterium prausnitzii for obesity-associated gut disorders. Front Pharmacol. (2021) 12:740636. doi: 10.3389/fphar.2021.740636 34925006 PMC8677946

[93] Tian F Zhang Y Liu H Wang X Chen L Zhao J . Effects of probiotic, prebiotic, and synbiotic supplementation on metabolic and inflammatory markers in type 2 diabetes mellitus: A systematic review and meta-analysis. Food Sci Hum Wellness. (2025) 14:1–15. doi: 10.26599/FSHW.2024.9250002

[94] Yang Y Yan J Li S Liu M Han R Wang Y . Efficacy of fecal microbiota transplantation in type 2 diabetes mellitus: a systematic review and meta-analysis. Endocrine. (2024) 84:48–62. doi: 10.1007/s12020-023-03606-1 38001323

[95] Xu YH Gao CL Guo HL Zhang WQ Huang W Tang SS . Sodium butyrate supplementation ameliorates diabetic inflammation in db/db mice. J Endocrinol. (2018) 238:231–44. doi: 10.1530/JOE-18-0137 29941502

[96] Chambers ES Byrne CS Morrison DJ Murphy KG Preston T Tedford C . Dietary supplementation with inulin-propionate ester or inulin improves insulin sensitivity in adults with overweight and obesity with distinct effects on the gut microbiota, plasma metabolome and systemic inflammatory responses: A randomised cross-over trial. Gut. (2019) 68:1430–8. doi: 10.1136/gutjnl-2019-318424 30971437 PMC6691855

[97] Choi Y Bose S Seo J Shin JH Lee D Kim Y . Effects of live and pasteurized forms of Akkermansia from the human gut on obesity and metabolic dysregulation. Microorganisms. (2021) 9:2039. doi: 10.3390/microorganisms9102039 34683361 PMC8538271

[98] Zheng L Ji YY Wen XL Duan SL . Fecal microbiota transplantation in the metabolic diseases: Current status and perspectives. World J Gastroenterol. (2022) 28:2546–60. doi: 10.3748/wjg.v28.i23.2546 35949351 PMC9254144

[99] Wu Z Zhang B Chen F Xia R Zhu D Chen B . Fecal microbiota transplantation reverses insulin resistance in type 2 diabetes: A randomized, controlled, prospective study. Front Cell Infect Microbiol. (2023) 12:1089991. doi: 10.3389/fcimb.2022.1089991 36704100 PMC9872724

[100] Wilson BC Vatanen T Cutfield WS O'Sullivan JM . The super-donor phenomenon in fecal microbiota transplantation. Front Cell Infect Microbiol. (2019) 9:2. doi: 10.3389/fcimb.2019.00002 30719428 PMC6348388

[101] Berry SE Valdes AM Drew DA Asnicar F Mazidi M Wolf J . Human postprandial responses to food and potential for precision nutrition. Nat Med. (2020) 26:964–73. doi: 10.1038/s41591-020-0934-0 32528151 PMC8265154

[102] Anwardeen NR Naja K Elrayess MA . Advancements in precision medicine: multi-omics approach for tailored metformin treatment in type 2 diabetes. Front Pharmacol. (2024) 15:1506767. doi: 10.3389/fphar.2024.1506767 39669200 PMC11634602

[103] Jiang R Zheng L Fang J Guan Q Yuan H Liang J . Targeting the gut microbiome for type 2 diabetes management: a scoping review of systematic reviews and meta-analyses. Front Endocrinol. (2026) 17:1682174. doi: 10.3389/fendo.2026.1682174 41694562 PMC12894762

[104] Ma Y Wang L Hu H Shieh AR Li E He D . Composition and function of gut microbiome: from basic omics to precision medicine. Genes. (2026) 17:116. doi: 10.3390/genes17010116 41595535 PMC12840750

[105] Tegegne HA Savidge TC . Gut microbiome metagenomics in clinical practice: bridging the gap between research and precision medicine. Gut Microbes. (2025) 17:2569739. doi: 10.1080/19490976.2025.2569739 41137523 PMC12562794

[106] Lin YT Sayols-Baixeras S Graells T Dekkers KF Baldanzi G Nguyen D . Identification of gut microbiome signatures and metabolites associated with albuminuria in type 2 diabetes. J Clin Endocrinol Metab. (2026) 111:e927–36. doi: 10.1210/clinem/dgaf453 40810199

[107] Zhou D Li S Hu G Wang Y Qi Z Xu X . Hypoglycemic effect of C. butyricum-pMTL007-GLP-1 engineered probiotics on type 2 diabetes mellitus. Gut Microbes. (2025) 17:2447814. doi: 10.1080/19490976.2024.2447814 39745177 PMC12931707

[108] Wang J Qu J Ye M Feng R Hui X Yang X . Beyond conventional therapies: gut microbiota modulation and macromolecular drugs in the battle against cardiometabolic diseases. J Pharm Anal. (2026) 16:101416. doi: 10.1016/j.jpha.2025.101416 41626567 PMC12856313

