# Correction: Gut microbiota in type 2 diabetes mellitus: mechanistic links between dysbiosis, insulin resistance, and chronic low-grade inflammation

**DOI:** 10.3389/fendo.2026.1899890

**Published:** 2026-06-15

**Authors:** Yi Chen, Danru Jin, Xue Han, Xiaoting Liu, Yisi Liu, Li Wang

**Affiliations:** 1 Department of Preventive Treatment of Disease, Affiliated Hospital of Liaoning University of Traditional Chinese Medicine, Liaoning, China; 2 Department of Cardiology I, The Affiliated Hospital of Liaoning University of Traditional Chinese Medicine, Liaoning, China

**Keywords:** bile acid signalling, branched-chain amino acid, gut dysbiosis, insulin resistance, microbial metabolites, short-chain fatty acids, type 2 diabetes mellitus

Author “Danru Jin” was erroneously omitted as equal contributing author.

The correct equal contribution statement is:

“†These authors contributed equally to this work and share first authorship.”

The original version of this article has been updated.

